# Risk Factors and Clinical Manifestations of Juxtacortical Small Lesions: A Neuroimaging Study

**DOI:** 10.3389/fneur.2017.00497

**Published:** 2017-09-22

**Authors:** Yilong Shan, Sha Tan, Yuge Wang, Kui Li, Lei Zhang, Siyuan Liao, Li Zhou, Zhezhi Deng, Xueqiang Hu, Haiyan Li, Xuejiao Men, Bingjun Zhang, Lisheng Peng, Zhuang Kang, Yan Zou, Zhengqi Lu

**Affiliations:** ^1^Department of Neurology, The Third Affiliated Hospital of Sun Yat-sen University, Guangzhou, China; ^2^Department of Physiatry, The Third Affiliated Hospital of Sun Yat-sen University, Guangzhou, China; ^3^Department of Neurology, The Fifth Affiliated Hospital of Sun Yat-sen University, Zhuhai, China; ^4^Department of Imaging, The Third Affiliated Hospital of Sun Yat-sen University, Guangzhou, China

**Keywords:** juxtacortical small lesions, magnetic resonance imaging, apolipoproteins B, homocysteine, headache

## Abstract

**Background and objective:**

White matter hyperintensities can be easily identified by brain imaging. Juxtacortical small lesion (JCSL) is a special type of white matter lesion, defined as no greater than 5 mm in diameter and adjacent to the cerebral cortex in location. We notice lately that JCSLs alone may be associated to various neurological symptoms. Here, we design the present study to determine the risk factors for JCSLs and their clinical manifestations in patients in our neurology clinic.

**Methods:**

206 participants suffered from neurological disorders and completed magnetic resonance imaging (MRI) examinations were divided into two groups: patients with JCSLs and patients without lesions on MRI. Meanwhile, 129 age- and sex-matched healthy volunteers were also recruited. Laboratory examinations and the phenotypes and distributions of the symptoms of the three groups were compared.

**Results:**

The serum levels of apoB and homocysteine (HCY) were independently related to the appearance of JCSLs and HCY level was also associated with the number of JCSLs. Patients with JCSLs might present with headache, insomnia, and/or anxiety/depression, which were related with the anatomical locations of the lesions.

**Conclusion:**

These data suggest that JCSLs are symptomatic and might in result fromarteriole atherosclerosis, which should raise our attention.

## Introduction

Brain imaging techniques are useful in the detection of different intracranial lesions and may help explain common neurological symptoms (1, 2). In the neurologic clinic, patients with chronic neurological symptoms such as headache and insomnia normally receive magnetic resonance imaging (MRI) for further diagnosis and treatment. Interestingly, part of these patients exhibit similar features on MRI, showing as one or more juxtacortical small lesions (JCSLs, <5 mm in diameter, within 10 mm from corticomedullary junction) (3), which were occasionally ignored by physicians because of the common symptoms. The neuroimaging characteristics of these lesions resembled those of white matter lesions (WMLs), which show high signal intensity on T2-weighted imaging (T2-WI) and low or equal signal intensity on T1-ted image (T1-WI) (1, 4).

In terms of locations, JCSLs belong to deep WMLs (DWMLs), which are classical categories of WMLs (5). But, JCSLs differ from conventional DWMLs because they are smaller in size and always appear independently in juxtacortex on neuroimage. It is well known that WMLs, especially DWMLs, represent a common cerebral small vessel disease (CSVD), and their presence is correlated with age, hypertension, carotid atherosclerosis, and diabetes mellitus (6–8). Additionally, chronic WMLs can also result in decreased cerebral blood flow and brain atrophy, causing cognitive decline or dementia (6, 7, 9, 10). Comparatively, JCSLs alone may also be symptomatic and it might be an early stage of CSVD. However, the relationships and differences between JCSLs and CSVD remain unclear. The correlative clinical features and risk factors of JCSLs need to be explored.

In the current study, we explored the risk factors and manifestations for JCSLs and compared them with the symptomatic patients without JCSLs and healthy volunteers.

## Materials and Methods

### Definition of JCSLs

Juxtacortical small lesions are defined as symptomatic lesions no greater than 5 mm in diameter and less than 10 mm from corticomedullary junction, with a well-demarcated high signal intensity on T2-WI and low or equal signal intensity on T1-WI. The hyperintense T2 lesion on juxtacortex is then confirmed on T2-fluid-attenuated inversion recovery (FLAIR) image with high signal intensity. The lesion is considered a definite JCSL if it is isointense on DWI, excluding an acute infarct lesion. JCSLs located in regions adjacent to the cortex were included in our study, while those in deep white matter regions involving the corona radiata, basal ganglia, internal capsule, thalamus, brainstem, or cerebellum were not considered.

### Subjects

**Figure 1:**
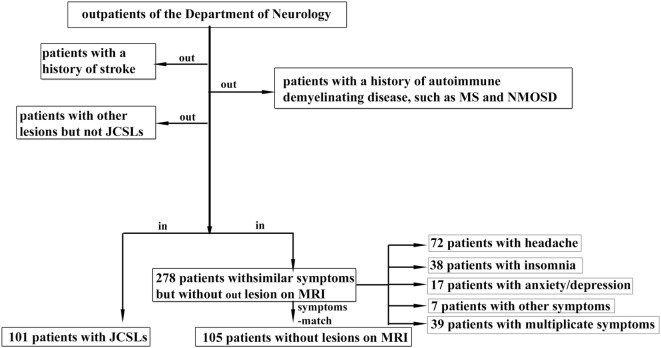
Patients inclusion chart. JCSLs, juxtacortical small lesions; MS, multiple sclerosis; NMOSD, neuromyelitis optica spectrum disorder; MRI, magnetic resonance imaging.

We conducted a retrospective study on patients who were admitted to the outpatient clinic of the Department of Neurology, the Third Affiliated Hospital of Sun Yat-sen University and also finished MRI scans, from January 2013 to September 2016 in the study. Patients matched one or more of the following conditions: (a) a history of stroke, (b) with diagnosed autoimmune demyelinating diseases, and (c) with other lesions on MRI, were excluded from the study. 101 individuals who had only JCSLs were finally recruited in our study. We also recruited 105 randomized and symptoms-matched patients from 278 outpatients with similar symptoms but without lesions on MRI in order to avoid the probability that symptom becoming a confounding factor. The inclusion procedure is presented in Figure 1. Besides, 129 age- and sex-matched volunteers (from the Medical Examination Center of the Third Affiliated Hospital of Sun Yat-Sen University) without any symptoms and histories of neurological diseases were also included as the control group. And, there was no abnormal finding in MRI of these volunteers. This study was approved by the ethics committee of the Third Affiliated Hospital of Sun Yat-sen University (No.2013-2-48) and all subjects gave written informed consent.

### Laboratory Analyses

Blood samples for fasting concentrations were collected from all patients in the morning after an overnight fast for >8 h. Based on the biomarkers of CSVD and related diseases, we tested the following laboratory parameters: homocysteine (HCY), glycated hemoglobin, 25 hydroxy vitamin D_3_ (25(OH)D_3_), total cholesterol, low-density lipoprotein cholesterol (LDL-C), high-density lipoprotein cholesterol, apolipoprotein-A (apoA), and apolipoprotein-B (apoB).

### Neuroimaging

Brain MRI scans was performed in all patients and healthy volunteers using a GE 3.0T MRI image scanner (General Electric, Milwaukee, WI, USA). The slice thickness of the axial scans ranged between 2 and 5 mm. Conventional MRI protocols were used in all patients, including T1 with and without gadolinium enhancement [400/15.5 ms, repetition time (TR)/echo time (TE)], T2 (2,500–3,500/100 ms, TR/TE), T2 (4,600–4,640/97.8–102 ms, TR/TE) fluid-attenuated inversion recovery (T2-FLAIR) (8,800/120 ms, TR/TE), diffusion-weighted imaging (DWI), and magnetic resonance angiography sequences.

### Assessment of Symptoms

Patients and volunteers underwent Mini-Mental State Examination (MMSE), with scores ≥26 considered normal cognition (11). The diagnosis of migraine without aura and tension-type headache fulfilled the criteria for migraine without aura and tension-type headache described by the International Headache Society (12). Patients with depression symptoms required to have had a Hamilton Depression Scale score of >7 while patients presented with anxiety symptoms was determined as score >7 on Hamilton Anxiety Rating Scale (HAM-A) (13). The diagnosis of primary insomnia was made according to the Chinese Classification and Diagnostic Criteria for Mental Disorders, third edition, which include: (1) difficulties in initiating or maintaining sleep, early morning awakening, and daytime dysfunction, (2) symptoms evident at least 3 days per week for at least 1 month, and (3) excluding any insomnia-related organic diseases. MMSE was used to evaluate the cognitive level of study people. All participants finished the above related assessments on the day collecting blood samples.

### Assessment of Risk Factors

Based on risks factors of CSVD and related diseases, we detected the following medical history of patients with JCSLs: hypertension, diabetes mellitus, and stroke. Data on demographic and medical history were collected by a detailed questionnaire. Data on medical history was obtained during the interview and were verified by medical records. The mean of two blood pressure measurements taken with a 5 min interval was calculated. Hypertension was defined as systolic blood pressure ≥140 mmHg and/or diastolic blood pressure ≥90 mmHg, or use of antihypertensive medication. Diabetes mellitus was defined as glycated hemoglobin ≥6.5% or use of antidiabetic drugs.

### Assessment of the JCSLs’ Location

The locations of the JCSLs by cerebral MRI were defined as the juxtacortex of the frontal lobe only (frontal lobe), juxtacortex of the parietal lobe only (parietal lobe), juxtacortex of both the frontal lobe and the parietal lobe (frontal parietal lobe), and both the frontal lobe and other regions according to the specific lobe of brain where the lesions appeared. And all the assessments were conducted in a blinded manner.

### Statistics

Comparisons between any two groups of patients with JCSLs, patients without JCSLs, and volunteers were performed using the Pearson χ^2^ test, Fisher exact test, independent samples *t* test or Mann–Whitney *U* test, as appropriate. To explore risk factors of JCSLs, factors with a *P* < 0.1 in univariate analysis were entered into multivariate logistic regression models (14). Linear regression was used to investigate the associations between numbers of JCSLs and factors (those with a *P* < 0.1 in univariate analysis) with adjustment for age, sex, MMSE score, HBP (yes vs no), and DM (yes vs no). Normality of the regression residuals was checked using histogram and normal probability plots of residuals. To examine the association between risk factors and different distributions of JCSLs or symptoms, different risk factors were compared among different distributions of JCSLs or symptoms by ANOVA, and if *P* < 0.05, a following post multiple comparison would carry out by using bonferroni or Tambane’s T2. SPSS 16.0 was used and α was set at 0.05 for all statistical analyses.

## Results

### Clinical Characteristics of the Study Populations

**Figure 2:**
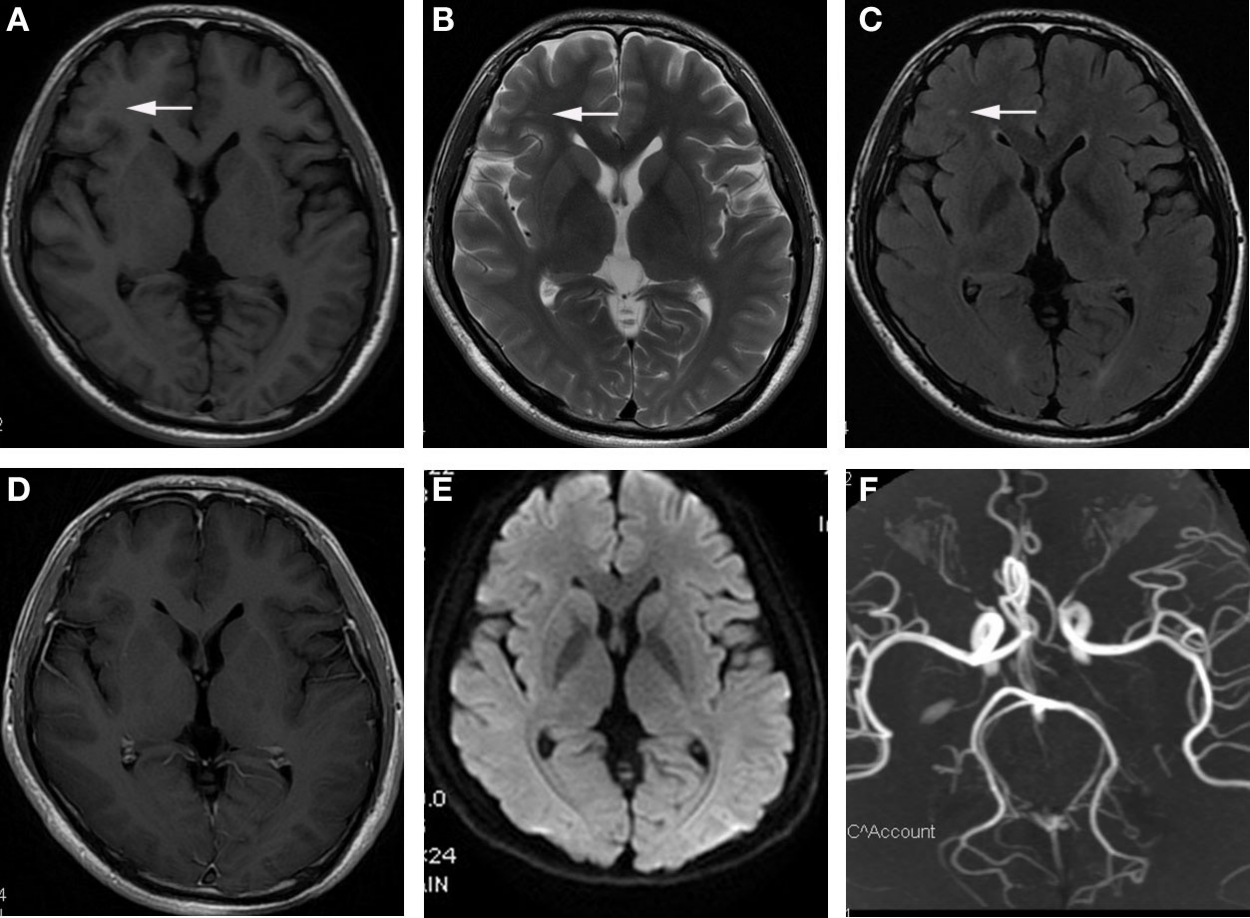
Representational serial magnetic resonance imaging findings of juxtacortical small lesion of a patient who complained about headache for 2 months. **(A)** low signal intensity on T1-weighted image (T1WI) in subcortical region of left frontal lobe (arrowhead); **(B,C)** high signal intensity on T2WI and T2 fluid attenuated inversion recovery sequences (arrowhead); **(D)** no enhancement on T1 + C image; **(E)** without abnormal signal in diffusion weighted imaging (DWI); **(F)** the normal image of magnetic resonance angiography (MRA).

101 patients recruited as patients with JCSLs and 105 symptoms-matched patients recruited as patients without lesions on MRI. There was no difference of the time interval between neuropsychological assessment and MRI scan in the comparison between patients with JCSLs and patients without JCSLs (6.57 ± 0.30 vs 6.18 ± 0.37, *P* = 0.412). Besides, 129 healthy volunteers were also recruited as control groups. Comparisons of baseline characteristics of patients with JCSLs (*n* = 101), patients without lesions on MRI (*n* = 105) and healthy controls are shown in Table 1. Of the 101 patients with JCSLs, symptoms principally presented as headache, insomnia, and anxiety/depression in 91 patients, while the remaining 10 patients experienced other untypical discomforts. Among those 91 patients, 41 patients (40.59%) suffered from headache (mainly migraine and tension-type headaches), 17 patients had sleep disorders, 9 had anxiety/depression, and the other 24 patients had two or more symptoms. Typical MRI images of the different sequences of JCSLs are shown in Figure 2.

**Table 1:** Clinical characteristics of study patients and healthy controls.

	Patients		Controls (*n* = 129)	P^a^		
	Patients with JCSLs	Patients without lesions on magnetic resonance imaging (MRI)		P_1_	P_2_	P_3_
Sex (male:female)	38:63	47:58	50:79	0.940	0.574	0.647
Age, years	50.62 ± 11.06	49.58 ± 10.65	48.10 ± 12.00	0.190	0.293	0.491
Symptoms, *n*	101	105	–	–	–	0.897^b^
Headache	41	46	–	–	–	–
Insomnia	17	19	–	–	–	–
Anxiety–depression	9	10	–	–	–	–
Other symptoms^c^	5	5	–	–	–	–
Multiplicate symptoms^d^	24	25	–	–	–	–
No symptoms	5	0	129	–	–	–
Serum 25(OH)D_3_	54.98 ± 22.08	60.05 ± 27.14	61.57 ± 18.97	**0.017**	0.814	0.090
Low-density lipoprotein cholesterol	3.05 ± 0.91	2.72 ± 0.71	2.75 ± 0.73	**0.006**	0.793	**0.006**
HDL-C	1.27 ± 0.28	1.35 ± 0.32	1.34 ± 0.40	0.185	0.816	0.187
ApoB	1.22 ± 0.42	1.06 ± 0.34	0.94 ± 0.29	**<0.001**	**0.005**	**0.004**
ApoA	1.42 ± 0.20	1.39 ± 0.19	1.37 ± 0.28	0.204	0.666	0.308
ApoB/apoA	0.89 ± 0.34	0.78 ± 0.29	0.71 ± 0.24	**<0.001**	**0.042**	**0.023**
HCY	12.44 ± 5.29	12.59 ± 7.92	10.8 ± 5.0	**0.020**	**0.040**	0.927
HBP	12	12	11	0.600	0.443	0.734
DM	7	9	7	0.347	0.232	0.887
MMSE score	29.39 ± 0.94	29.44 ± 0.77	29.38 ± 0.93	0.960	0.789	0.704
Time interval (days)^e^	6.57 ± 0.30	6.18 ± 0.37	0 ± 0	**<0.001**	**<0.001**	0.412

*JCSLs, juxtacortical small lesions; 25(OH)D_3_, 25-hydroxyvitamin D_3_; LDL, low-density lipoprotein; HDL, high-density lipoprotein; apoB, apolipoprotein B; apoA, apolipoprotein A; HCY, homocysteine; HBP, hypertension; DM, diabetes mellitus; MMSE, Mini-Mental State Examination; *P, P*-value; P_1_, Patients with JCSLs vs healthy controls; P_2_, Patients without JCSLs vs healthy controls; P_3_, Patients with JCSLs vs Patients without JCSLs*.

*^a^Independent samples t-test or Mann–Whitney U-test to complete the comparisons between each two groups*.

*^b^The P-value of the distribution of symptoms between patients with or without JCSLs*.

*^c^Other symptoms included limb weakness, tremor, and vertigo*.

*^d^Multiplicate symptoms included two or more types of symptoms mentioned all above*.

*^e^Time interval between neuropsychological assessments and MRI scan*.

*The bold font of numbers refer to P < 0.05*.

### Independent Predictors of JCSLs

As shown in Table 1, the presence of JCSLs was related to serum 25(OH)D_3_ (*P* = 0.017), LDL-C (*P* = 0.006), apoB (*P* < 0.001), apoB/apoA (*P* < 0.001), or HCY (*P* = 0.020) when patients with JCSLs compared with healthy controls while JCSLs was related to LDL-C (*P* = 0.006), apoB (*P* = 0.004), apoB/apoA (*P* = 0.023) when patients with JCSLs compared with patients without lesions. In logistic regression models (Table 2), the presence of JCSLs were still related to apoB no matter patients with JCSLs compared with healthy controls [odds ratio (OR), 19.648, 95% confidence interval (CI), 2.693–143.379; *P* = 0.003] or patients without lesions (OR, 2.947; 95% CI, 1.010–8.797; *P* = 0.047). Further adjustment for age, gender, HBP, DM, and MMSE score did not change the above statistical differences (*P* = 0.001, *P* = 0.022, respectively). In addition, the logistic regression model also revealed HCY was associated with JCSLs in the comparison of patients with JCSLs and healthy controls (OR, 1.061; 95% CI, 1.001–1.125; *P* = 0.048).

**Table 2:** Logistic regression analysis to identify risk factors independently associated with JCSLs.

	OR (95% CI)	*P*_1_	*P*_2_^a^
**Factors associated with JCSLs when comparing with healthy controls**			
Serum 25(OH)D_3_	0.991 (0.976–1.007)	0.257	0.223
LDL-C	0.949 (0.604–1.492)	0.821	0.684
ApoB	19.648 (2.693–143.379)	**0.003**	**0.001**
ApoB/apoA	0.435 (0.047–4.061)	0.465	0.292
Homocysteine	1.061 (1.001–1.125)	**0.048**	**0.012**
**Factors associated with JCSLs when comparing with patients without lesions**			
Serum 25(OH)D_3_	0.994 (0.981–1.006)	0.335	0.229
LDL-C	1.404 (0.927–2.127)	0.109	0.103
ApoB	2.967 (1.010–8.797)	**0.047**	**0.022**
ApoB/apoA	0.462 (0.032–4.774)	0.391	0.313

*JCSLs, juxtacortical small lesions; OR, odds ratio; 95% CI, 95% confidence interval; LDL-C, low-density lipoprotein; apoB, apolipoprotein B; apoA, apolipoprotein A; 25(OH)D_3_, 25-hydroxyvitamin D_3_; HBP, hypertension; DM, diabetes mellitus; MMSE, Mini-Mental State Examination*.

*^a^P_2_ further adjustment for age, sex, DM, HBP, and MMSE score*.

*The reference of sex, DM, and HBP categories are man, no DM, and no HBP relatively*.

*The bold font of numbers refer to P < 0.05*.

### Relationship between the Number of JCSLs and Risk Factors

The association between risk factors and the number of JCSLs was evaluated by Linear regression as shown in Table 3. The number of JCSLs was only significantly positive related with HCY (β = 0.103; 95% CI,0.021–0.185; *P* = 0.015 after adjusting for age, sex, history of HBP, history of DM, and MMSE score). Further adjustments of other risk factors did not change this association (β = 0.096; 95% CI, 0.009–0.183; *P* = 0.031).

**Table 3:** Linear regression analysis to identify the association between risk factors and the number of JCSLs.

Variable	Crude β (95% CI)	*P*	Adjusted β (95% CI)^a^	*P*
Serum 25(OH)D_3_	0.004 (−0.015–0.023)	0.687	−0.003 (−0.022–0.016)	0.733
Low-density lipoprotein cholesterol	0.172 (−0.289–0.633)	0.461	0.120 (−0.345–0.585)	0.610
ApoB	0.446 (−0.566–1.457)	0.384	0.406 (−0.634–1.446)	0.440
ApoB/apoA	0.256 (−1.025–1.537)	0.692	0.230 (−1.080–1.541)	0.728
HCY	0.094 (0.013–0.175)	**0.023**	0.103 (0.021–0.185)	**0.015**

*JCSLs, juxtacortical small lesions; ρ, correlation coefficent; *P, P* value; 25(OH)D_3_, 25-hydroxyvitamin D_3_; LDL, low-density lipoprotein; HDL, high-densitylipoprotein apoB, apolipoprotein B; apoA, apolipoprotein A; HCY, homocysteine*.

*^a^Adjusted for age, sex, DM, HBP, and Mini-Mental State Examination score*.

*The reference of sex, DM, and HBP categories are man, no DM, and no HBP relatively*.

*The bold font of numbers refer to P < 0.05*.

### Phenotypic Symptoms of JCSLs and the Relationship between Symptoms and JCSL Distributions

**Figure 3:**
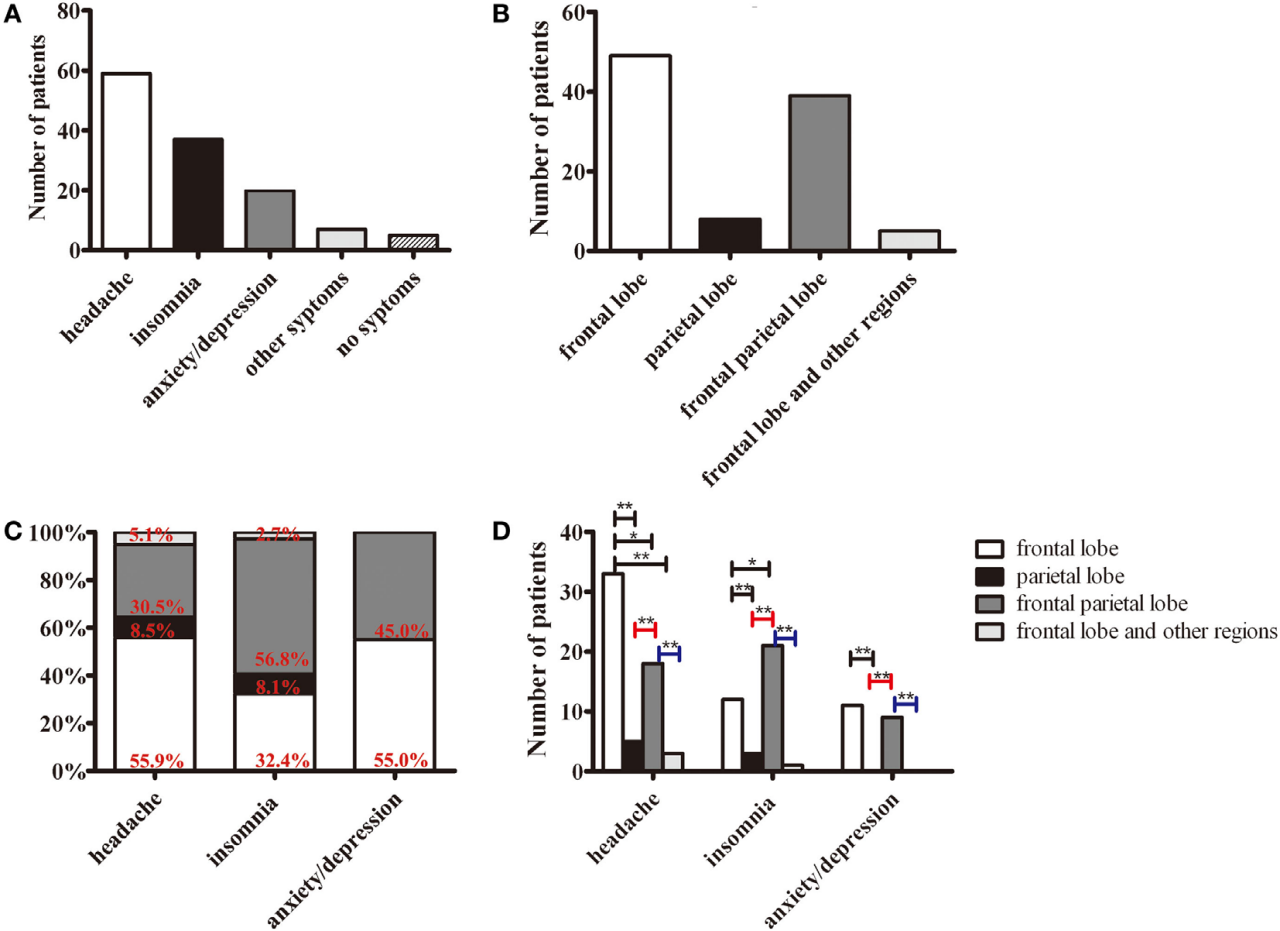
The distributions of symptoms and juxtacortical small lesions (JCSLs). **(A)** The classification of symptoms in patients with JCSLs; **(B)** the distribution of JCSLs; **(C)** the distribution of JCSLs in different symptoms shown in a percentage bar graph; **(D)** the association between the distribution of JCSLs and different symptoms evaluated using Pearson χ^2^ test. *P < 0.05, **P < 0.01.

**Figure 4:**
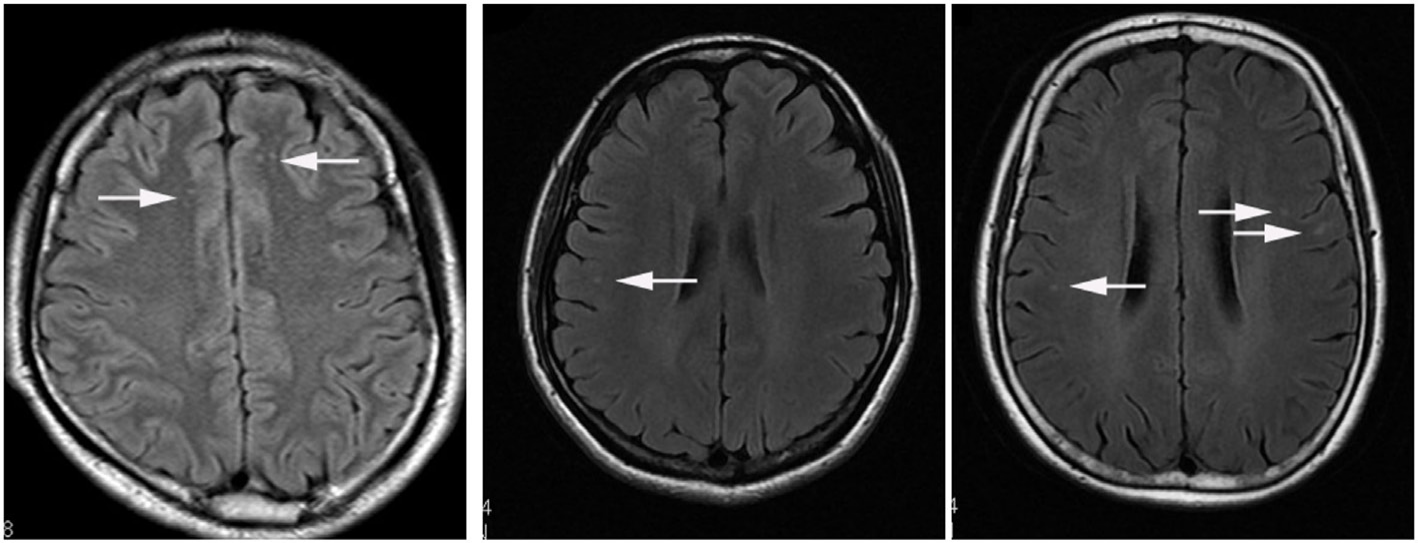
The axial view of typical presentations of different distributions of juxtacortical small lesion/s (JCSL/s) on T2 FLAIR. **(A)** JCSLs in juxtacortical region of frontal lobe (arrowhead); **(B)** JCSL in juxtacortical region of right parietal lobe (arrowhead); **(C)** JCSLs in subcortical region of frontal parietal lobe (arrowhead).

The major symptoms in patients with JCSLs were headache (59 patients, 58.4%), insomnia (37 patients, 36.6%), and anxiety/depression (20 patients, 19.8%) (Figure 3A). JCSLs were mainly distributed in the frontal lobe (49 patients, 48.5%) and frontal parietal lobe (39 patients, 38.6%) (Figures 3B and 4A–C). As shown in Figures 3C,D, the location of JCSLs was more likely to be only frontal lobe in patients suffering headache (vs frontal parietal lobe, *P* < 0.05, Pearson χ^2^ test) while the percentage of JCSLs presenting in frontal parietal lobe was the largest in patients with insomnia (vs frontal lobe, *P* < 0.05, Pearson χ^2^ test). In addition, patients with anxiety–depression had a similar distribution of JCSLs in the frontal lobe or frontal parietal lobe (frontal lobe vs frontal parietal lobe, *P* > 0.05).

### Relationship between the Distribution of JCSLs/Symptoms and Risk Factors

Because of the difference of laboratory findings found in the comparison of patients with JCSLs and healthy controls, we further used ANOVA to compare the levels of risk factors in different distribution of JCSLs or symptoms. As shown in Table S1 in Supplementary Material, the distribution of JCSLs and different symptoms had no statistical association with any risk factors.

## Discussion

The main finding of the present study was that the presence of JCSLs was related to higher serum levels of apoB and HCY. Additionally, although patients with symptoms with or without JCSLs showed similar HCY levels, there was a positive correlation between the HCY levels and the number of lesions. Moreover, the various symptom categories of the patients were related to the anatomical locations of the JCSLs.

In the present study, the levels of apoB and HCY were both the independent risk factors of JCSLs. To date, as part of LDL, elevated serum apoB levels, especially the apoB/apoA ratio, were previously reported to contribute to atherosclerosis (15). Furthermore, the apoB/apoA ratio was recently recognized as the best predictor for atherosclerosis among all cholesterol indicators (15). Besides, HCY also promotes atherosclerosis formation through stimulating the production of sulfated glycosaminoglycans, which is the key component of atherosclerotic plaque (16). Interestingly, it was HCY but not apoB or apoB/apoA that positive related to the number of JCSLs, indicating that high apoB levels induced JCSLs but HCY exacerbated the process of JCSLs. Previous studies have pointed out that a dose-dependent HCY combine with LDL can lead to an excessive production of reactive oxygen species, which then accelerate the process of atherosclerosis (17). In addition, DWMLs are proved to be consistent with small vessel disease (18). Taken together, we consider that JCSLs might result from the atherosclerotic blockage of arteriole in juxtacortex and may also represent an early stage of CSVD. However, the precise mechanism remains unknown, although it may involve microemboli from atherosclerotic arteriole or local arteriole atheromatous plaque formation.

Metabolic syndrome, hypertension, diabetes mellitus, and hyperhomocysteinemia are the classical risk factors for WMLs and CSVD (19, 20), but the risk factors of JCSLs were less complex. It is worth mentioned that patients with WMLs are usually elder people with the age ranges from 65 to 80 and the severity of WMLs increases with age (2, 19–21). But based on our study, the average age of patients with JCSLs was about 50, which was much younger than that of patients with WMLs. Maybe this is one of the reasons why patients with JCSLs get less positive biomarkers and risk factors. And we realized that the high level of serum apoB (or apoB/apoAI ratio, which is part of metabolic syndrome) and HCY are both related to JCSLs and WMLs (17, 22), suggesting they could be two stages of CSVD and JCSLs that may continue to progress if left untreated.

Although the sizes of JCSLs were small, they still determined corresponding clinical symptoms. Despite the predominance location of JCSLs in the frontal lobe, the various symptoms in our patients were associated with the distribution of JCSLs. For example, in patients with headache, the JCSLs were more likely to appear in the frontal lobe. Similarly, WMLs in patients with migraine and tension-type headache were reported to be mainly localized in the frontal lobe (23). We also found that patients with depression or anxiety exhibited lesions in both the frontal and parietal lobes. In support, lower glucose metabolism was found in both frontal and parietal lobe in patients with depression and anxiety (24, 25). Furthermore, the severity of anxiety was proved to be negative related with the level of choline/N-acetylaspartate in prefrontal lobe (26). On the other hand, the punctuate, but not large DWMLs (<3 mm) of elderly subjects with a history of major depression was more likely to be ischemic lesions than that of controls in a pathological study of cerebral tissues (27). Finally, we found that patients with insomnia were most likely to show lesions in the frontal parietal lobe. Nevertheless, previous studies suggested that insomnia was related to changes in the global cerebral arousal system, rather than local changes (28), and that insomnia patients have altered neurotransmitter levels, including gamma-aminobutyric acid, glutamate, and 5-hydroxytryptamine (29). Lesions on white matter can influence the neurotransmitter level in brain, which may lead to critical illness besides of common symptoms. One study suggested that severe WMLs could eventually cause dementia, which can be improved by increasing the level of acetylcholine in brain (30). Speculatively, with local JCSLs, degeneration of regional axon nerve fiber tracts may result in abnormal corresponding neurotransmitter signals and abnormal neurotransmitter secretion, eventually leading to different symptoms including headache, depression, anxiety, and insomnia (29, 31). These results remind us that when dealing with patients with common symptoms, it is necessary to explore potential risk factors and neuroimaging changes for the purpose of avoiding worsening diseases.

We found that JCSLs were predominantly distributed in the frontal lobe and the frontal parietal lobe. This vulnerability of these regions may be related to the relatively poor blood supply of the anterior circulation. A recent study in young adults reported that large-artery atherosclerosis was more frequent in patients with anterior circulation stroke than in those with posterior circulation stroke (32), which was partly consistent with our result. Moreover, juxtacortical white matter, consists of U-fibers instead of long white matter tracts, is mostly supplied by short vessels at the boundary of white matter and cortex or the end of long penetrating medullary vessels, which would be easily influenced by atherosclerosis (3, 33). Younger age and special blood supply may be explanations for this phenomenon, but future high-resolution MRI studies are required to draw a conclusion.

A potential limitation of our study is a single-center design involved a modest sample size of Chinese patients in a neurology clinic. Limiting to the small sample, the unbalanced distribution of symptoms or lesions locations may lead to some significant conclusion lost. And multi-center studies with large and diverse cohorts, as well as ethnicities, should be performed in the future. Furthermore, the retrospective design study did not allow us to determine the time interval or order between the appearance of imaging findings and the patients’ complaints, making it difficult to determine causality. Moreover, the time interval between neuropsychological assessments and MRI scan may affect the accuracy of the data. Finally, further high resolution MRI studies and prospective cohort studies are required to confirm our findings.

In conclusion, we found that the elevated apoB and HCY level were the independent predictors of JCSLs, indicating that arteriole atherosclerosis may be causative in the disease process. And based on our results, with younger age of onset and less risk factors, JCSLs may be an early stage of WMLs. A variety of chronic clinical neurological symptoms can help us earlier discover and deal with JCSLs.

## Author Contributions

YS, ST, and ZL: acquisition of data and drafting of the manuscript. YS, ST, YW, and KL: acquisition of data. LZ, SL, LiZ, ZD, XH, HL, and XM: statistical analysis. BZ, LP, and ZK: revising the manuscript. ZL and YZ: conceiving the study and drafting of the manuscript.

## Conflict of Interest Statement

The authors declare that the research was conducted in the absence of any commercial or financial relationships that could be construed as a potential conflict of interest.

## References

[1] de Leeuw FE de Groot JC Achten E Oudkerk M Ramos LM Heijboer R Prevalence of cerebral white matter lesions in elderly people: a population based magnetic resonance imaging study. The Rotterdam Scan Study. J Neurol Neurosurg Psychiatry (2001) 70(1):9–14. 10.1136/jnnp.70.1.9 11118240 PMC1763449

[2] Moroni F Ammirati E Magnoni M D’Ascenzo F Anselmino M Anzalone N Carotid atherosclerosis, silent ischemic brain damage and brain atrophy: a systematic review and meta-analysis. Int J Cardiol (2016) 223:681–7. 10.1016/j.ijcard.2016.08.234 27568989

[3] Moody DM Bell MA Challa VR. Features of the cerebral vascular pattern that predict vulnerability to perfusion or oxygenation deficiency: an anatomic study. AJNR Am J Neuroradiol (1990) 11(3):431–9. 2112304 PMC8367475

[4] Ylikoski A Erkinjuntti T Raininko R Sarna S Sulkava R Tilvis R. White matter hyperintensities on MRI in the neurologically nondiseased elderly. Analysis of cohorts of consecutive subjects aged 55 to 85 years living at home. Stroke (1995) 26(7):1171–7. 10.1161/01.STR.26.7.1171 7604409

[5] Fazekas F Chawluk JB Alavi A Hurtig HI Zimmerman RA. MR signal abnormalities at 1.5 T in Alzheimer’s dementia and normal aging. AJR Am J Roentgenol (1987) 149(2):351–6. 10.2214/ajr.149.2.351 3496763

[6] Debette S Markus HS. The clinical importance of white matter hyperintensities on brain magnetic resonance imaging: systematic review and meta-analysis. BMJ (2010) 341:c3666. 10.1136/bmj.c3666 20660506 PMC2910261

[7] Pantoni L. Cerebral small vessel disease: from pathogenesis and clinical characteristics to therapeutic challenges. Lancet Neurol (2010) 9(7):689–701. 10.1016/S1474-4422(10)70104-6 20610345

[8] Manolio TA Burke GL O’Leary DH Evans G Beauchamp N Knepper L Relationships of cerebral MRI findings to ultrasonographic carotid atherosclerosis in older adults: the Cardiovascular Health Study. CHS Collaborative Research Group. Arterioscler Thromb Vasc Biol (1999) 19(2):356–65. 10.1161/01.ATV.19.2.356 9974419

[9] Appelman AP Vincken KL van der Graaf Y Vlek AL Witkamp TD Mali WP White matter lesions and lacunar infarcts are independently and differently associated with brain atrophy: the SMART-MR study. Cerebrovasc Dis (2010) 29(1):28–35. 10.1159/000255971 19893309

[10] Vernooij MW van der Lugt A Ikram MA Wielopolski PA Vrooman HA Hofman A Total cerebral blood flow and total brain perfusion in the general population: the Rotterdam Scan Study. J Cereb Blood Flow Metab (2008) 28(2):412–9. 10.1038/sj.jcbfm.9600526 17622253

[11] Tombaugh TN McIntyre NJ. The mini-mental state examination: a comprehensive review. J Am Geriatr Soc (1992) 40(9):922–35. 10.1111/j.1532-5415.1992.tb01992.x 1512391

[12] Eriksen MK Thomsen LL Olesen J. New international classification of migraine with aura (ICHD-2) applied to 362 migraine patients. Eur J Neurol (2004) 11(9):583–91. 10.1111/j.1468-1331.2004.00890.x 15379737

[13] Ionescu DF Niciu MJ Henter ID Zarate CA. Defining anxious depression: a review of the literature. CNS Spectr (2013) 18(5):252–60. 10.1017/S1092852913000114 23507190 PMC3773528

[14] Wang Z van Veluw SJ Wong A Liu W Shi L Yang J Risk factors and cognitive relevance of cortical cerebral microinfarcts in patients with ischemic stroke or transient ischemic attack. Stroke (2016) 47(10):2450–5. 10.1161/STROKEAHA.115.012278 27539302

[15] Sniderman AD Furberg CD Keech A Roeters van Lennep JE Frohlich J Jungner I Apolipoproteins versus lipids as indices of coronary risk and as targets for statin treatment. Lancet (2003) 361(9359):777–80. 10.1016/S0140-6736(03)12663-3 12620753

[16] Casado-Naranjo I Romero Sevilla R Portilla Cuenca JC Duque de San Juan B Calle Escobar ML Fernandez Pereira L Association between subclinical carotid atherosclerosis, hyperhomocysteinaemia and mild cognitive impairment. Acta Neurol Scand (2016) 134(2):154–9. 10.1111/ane.12525 26503595

[17] Zinellu A Sotgia S Scanu B Pintus G Posadino AM Cossu A S-homocysteinylated LDL apolipoprotein B adversely affects human endothelial cells in vitro. Atherosclerosis (2009) 206(1):40–6. 10.1016/j.atherosclerosis.2009.01.035 19249051

[18] Read SJ Pettigrew L Schimmel L Levi CR Bladin CF Chambers BR White matter medullary infarcts: acute subcortical infarction in the centrum ovale. Cerebrovasc Dis (1998) 8(5):289–95. 10.1159/000015868 9712927

[19] Kuo HK Chen CY Liu HM Yen CJ Chang KJ Chang CC Metabolic risks, white matter hyperintensities, and arterial stiffness in high-functioning healthy adults. Int J Cardiol (2010) 143(2):184–91. 10.1016/j.ijcard.2009.02.005 19261342

[20] Launer LJ. Epidemiology of white matter lesions. Top Magn Reson Imaging (2004) 15(6):365–7. 10.1097/01.rmr.0000168216.98338.8d 16041288

[21] van Veluw SJ Zwanenburg JJ Engelen-Lee J Spliet WG Hendrikse J Luijten PR In vivo detection of cerebral cortical microinfarcts with high-resolution 7T MRI. J Cereb Blood Flow Metab (2013) 33(3):322–9. 10.1038/jcbfm.2012.196 23250109 PMC3587820

[22] Ying X Qian Y Jiang Y Jiang Z Song Z Zhao C. Association of the apolipoprotein B/apolipoprotein A-I ratio and low-density lipoprotein cholesterol with insulin resistance in a Chinese population with abdominal obesity. Acta Diabetol (2012) 49(6):465–72. 10.1007/s00592-012-0419-9 22965469

[23] De Benedittis G Lorenzetti A Sina C Bernasconi V. Magnetic resonance imaging in migraine and tension-type headache. Headache (1995) 35(5):264–8. 10.1111/j.1526-4610.1995.hed3505264.x 7775189

[24] Biver F Goldman S Delvenne V Luxen A De Maertelaer V Hubain P Frontal and parietal metabolic disturbances in unipolar depression. Biol Psychiatry (1994) 36(6):381–8. 10.1016/0006-3223(94)91213-0 7803599

[25] Greicius MD Flores BH Menon V Glover GH Solvason HB Kenna H Resting-state functional connectivity in major depression: abnormally increased contributions from subgenual cingulate cortex and thalamus. Biol Psychiatry (2007) 62(5):429–37. 10.1016/j.biopsych.2006.09.020 17210143 PMC2001244

[26] Moon CM Kang HK Jeong GW. Metabolic change in the right dorsolateral prefrontal cortex and its correlation with symptom severity in patients with generalized anxiety disorder: proton magnetic resonance spectroscopy at 3 Tesla. Psychiatry Clin Neurosci (2015) 69(7):422–30. 10.1111/pcn.12279 25611853

[27] Fazekas F Kleinert R Offenbacher H Schmidt R Kleinert G Payer F Pathologic correlates of incidental MRI white matter signal hyperintensities. Neurology (1993) 43(9):1683–9. 10.1212/WNL.43.9.1683 8414012

[28] Roth T Insomnia: definition, prevalence, etiology, and consequences. J Clin Sleep Med (2007) 3(5 Suppl):S7–10. 17824495 PMC1978319

[29] Zhang R Yang YS Liu XC Yang JL Li YH Shi PZ Correlation study of basic Chinese medicine syndromes and neurotransmitter levels in patients with primary insomnia. Chin J Integr Med (2016). 10.1007/s11655-016-2752-2 28028724

[30] Gudmundsson P Olesen PJ Simoni M Pantoni L Östling S Kern S White matter lesions and temporal lobe atrophy related to incidence of both dementia and major depression in 70-year-olds followed over 10 years. Eur J Neurol (2015) 22(5):781–8. 10.1111/ene.12651 25598324

[31] Butt AM Fern RF Matute C. Neurotransmitter signaling in white matter. Glia (2014) 62(11):1762–79. 10.1002/glia.22674 24753049

[32] von Sarnowski B Schminke U Grittner U Tanislav C Bottcher T Hennerici MG Posterior versus anterior circulation stroke in young adults: a comparative study of stroke aetiologies and risk factors in stroke among young Fabry patients (sifap1). Cerebrovasc Dis (2017) 43(3–4):152–60. 10.1159/000454840 28088807

[33] Rowbotham GF Little E A new concept of the circulation and the circulations of the brain. The discovery of surface arteriovenous shunts. Br J Surg (1965) 52:539–42. 10.1002/bjs.1800520714 14315697

