# Supplementary material for: Risk Factors and Clinical Manifestations of Juxtacortical Small Lesions: A Neuroimaging Study
Source: Front Neurol. 2017 Sep 22;8:497. doi: 10.3389/fneur.2017.00497 (PMC5614934; doi:10.3389/fneur.2017.00497)
Supplement: Supplementary file 1 [file Table_1.DOCX]

**Supplement Table 1. Difference of serum25(OH)D_3_, LDL-C, ApoB, ApoB/apoA, HCY among different distributions of juxtacortical small lesions (A) or symptoms (B) through using ANOVA analysis.**

(A)

|  | Distribution of JCSLs | | | | **P** |
| --- | --- | --- | --- | --- | --- |
|  | FL | PL | FPL | FLOR |  |
| Serum25(OH)D_3_ | 55.34 ± 24.57 | 48.06 ± 13.13 | 56.37 ± 20.69 | 49.95 ± 17.92 | 0.793 |
| LDL-C | 2.96 ± 0.92 | 3.25 ± 0.91 | 3.09 ± 0.89 | 3.28 ± 1.14 | 0.762 |
| ApoB | 1.16 ± 0.43 | 1.29 ± 0.24 | 1.26 ± 0.44 | 1.43 ± 0.39 | 0.438 |
| ApoB/apoA | 0.86 ± 0.37 | 0.96 ± 0.26 | 0.88 ± 0.31 | 1.02 ± 0.32 | 0.729 |
| HCY | 12.03 ± 5.31 | 12.79 ± 3.83 | 13.15 ± 5.71 | 10.62 ± 4.04 | 0.680 |
|  |  |  |  |  |  |

**Abbreviations:**JCSLs, juxtacortical small lesions; FL, frontal lobe; PL, parietal lobe; FPL, frontal parietal lob; FLOR, frontal lobe and other regions;25(OH)D_3_, 25-hydroxyvitamin D_3_; LDL, low-density lipoprotein; HDL, high-densitylipoprotein apoB, apolipoprotein B; apoA, apolipoprotein A; HCY, homocysteine; P, P-value.

(B)

|  | Symptoms | | | | | | **P** |
| --- | --- | --- | --- | --- | --- | --- | --- |
|  | headache | insomnia | anxiety-  depression | other symptoms | Mix  Symptoms | no symptoms |  |
| 25(OH)D_3_ | 50.97±22.50 | 65.30± 22.97 | 53.89 ± 17.51 | 52.88 ± 22.82 | 55.95 ± 21.44 | 56.22 ± 21.31 | 0.295 |
| LDL-C | 3.02 ± 1.04 | 3.23 ± 0.80 | 2.91 ± 0.51 | 2.86 ± 0.76 | 3.10 ± 0.95 | 3.12 ± 0.88 | 0.859 |
| ApoB | 1.26 ± 0.52 | 1.33 ± 0.36 | 1.09 ± 0.31 | 1.12 ± 0.39 | 1.17 ± 0.33 | 1.14 ± 0.34 | 0.550 |
| ApoB/apoA | 0.92 ± 0.40 | 0.95 ± 0.25 | 0.81 ± 0.24 | 0.82 ± 0.35 | 0.83 ± 0.30 | 0.88 ± 0.37 | 0.710 |
| HCY | 12.88±5.45 | 11.93 ± 4.85 | 11.79 ± 6.59 | 13.43 ± 5.30 | 11.88 ± 5.12 | 11.23 ± 5.33 | 0.893 |

**Abbreviations:**25(OH)D_3_, 25-hydroxyvitamin D_3_; LDL, low-density lipoprotein; HDL, high-densitylipoprotein apoB, apolipoprotein B; apoAI, apolipoprotein AI; HCY, Homocysteine; P, P-value.
